# A novel true triaxial apparatus for high-stress low-frequency disturbance in hard rocks: Development, validation, and application

**DOI:** 10.1371/journal.pone.0324033

**Published:** 2025-05-28

**Authors:** Chao Peng, Hanwen Jia, Yang Liu, Huanxin Liu, Xiwei Zhang

**Affiliations:** 1 Deep Mining Laboratory of Shandong Gold Group Co., Ltd., Laizhou, Shandong, China; 2 Shandong Gold Mining Co., Ltd., Jinan, China; 3 Key Laboratory of Ministry of Education on Safe Mining of Deep Metal Mines, Northeastern University, Shenyang, Liaoning, China; University of Science and Technology Beijing, CHINA

## Abstract

A novel true triaxial apparatus (TTA) has been designed and fabricated to investigate the mechanical behavior of deep underground engineering under high-stress conditions and low-frequency disturbance loads. This apparatus features a two-rigid, one-flexible loading system, with rigid loading applied along the directions of the maximum and intermediate principal stresses, offering maximum load capacities of 2000 kN and 4000 kN, respectively. The direction of the maximum principal stress is also equipped with dynamic loading capabilities, enabling low-frequency disturbance loads with frequencies up to 20 Hz and amplitudes of 0.5 mm. The minimum principal stress direction utilizes flexible loading, with pressure capabilities of up to 120 MPa. Moreover, the integration of a high-rigidity loading frame and high-precision servo control systems has significantly enhanced the apparatus’s performance and data accuracy, particularly in small-scale deformation tests. Additionally, a dual-actuator, dual-loop servo control mode is employed to effectively suppress eccentric loading effects in true triaxial tests. To validate the reliability of the TTA and to preliminarily explore the effects of stress paths and disturbances on deep rock mechanical properties, true triaxial tests were conducted using granite. The results demonstrate that both the intermediate principal stress and disturbance frequency significantly influence the strength and failure modes of the rock. Static and disturbance tests exhibited excellent high repeatability and consistency, further confirming the accuracy and reliability of the apparatus. Overall, the TTA provides a novel methodology for investigating the mechanical properties of deep rock masses under high-stress and low-frequency disturbance conditions, making it an effective tool for addressing related scientific and engineering challenges.

## 1. Introduction

The growing demand for metals driven by global economic growth, coupled with the depletion of near-surface mineral deposits, is pushing mineral extraction to greater depths [1–4]. For example, many gold mines in South Africa and Canada now operate at depths exceeding 2000 meters. The TauTona Gold Mine, or instance, has reached a depth of 3500 meters [5], while the Mponeng Gold Mine has surpassed a depth of 4000 meters [6], making it the deepest mine in the world to date. In Canada, the Creighton Nickel Mine in Sudbury, Ontario, operates at a depth of 2255 meters [7], and the Kidd Creek Mine in Timmins has achieved a mining depth of 3000 meters [8]. Similarly, China hosts over 40 metal mines with depths exceeding 1000 meters [9–11]. Notable examples include the Hongtoushan Copper Mine, which reaches a depth of 1600 meters, as well as the Sanshandao Gold Mine, the Jiapigou Gold Mine, and the Huize Lead-Zinc Mine, each surpassing the 1000-meter mark.

The extraction of deep mineral resources encounters extremely challenging geomechanical conditions characterized by high in-situ stresses and engineering-induced disturbances. These factors play a critical role in triggering rock mechanics problems and geological hazards in deep mining operations [12–14]. Conventional static experimental studies on rock mechanical behavior under triaxial stress states (*σ*_1_ > *σ*_2_ = *σ*_3_) are inadequate for addressing the complexities of deep engineering projects. Consequently, the development of advanced true triaxial apparatuses with dynamic loading capabilities is imperative. Such systems facilitate the investigation of brittle fracture mechanisms and the dynamic instability of rocks under high-stress conditions, driving significant advancements in geotechnical and mining engineering. These innovations are particularly valuable for mitigating major challenges in deep engineering, including the prevention and control of geological disasters [15–18].

To investigate the mechanical properties of rocks under actual in-situ stress conditions (*σ*_1_ > *σ*_2_ > *σ*_3_), researchers have developed various types of true triaxial apparatus [19]. Based on the applied stress boundary conditions, three typical techniques are commonly used to achieve true triaxial stress loading. (a) Rigid loading: all three principal stresses are applied using rigid indenters to replicate true triaxial stress boundary conditions. (b) Flexible loading: flexible materials, such as pressure pads, hydraulic systems, or liquid-filled capsules, are used to apply stresses. These systems typically have lower loading capacities and are primarily suitable for testing soils and soft rocks. (c) Hybrid loading: a representative example is Mogi’s “two rigid and one flexible” device, where two rigid systems apply the maximum and intermediate principal stresses, and a flexible system applies the minimum principal stress. The following section provides a concise overview of several exemplary true triaxial apparatuses.

(1) Hydraulic quasistatic true triaxial apparatus

Mogi [20] from the University of Tokyo pioneered the development of the world’s first true triaxial rock testing apparatus, which unveiled the critical role of the intermediate principal stress in rock deformation and strength. This landmark discovery has catalyzed the advancement and ongoing refinement of various true triaxial loading test instruments [21–25]. Haimson [26] developed a servo-functional true triaxial rock testing apparatus, which effectively addressed challenges such as volumetric deformation measurement and post-peak servo control. However, due to limitations in deformation measurement sensors, only partial post-peak strength curves for hard rocks could be obtained. Despite this, the research findings have been successfully applied in geophysical and earthquake-related projects [27–29]. Wei You [30] of Sichuan University designed a true triaxial rock loading system to investigation of the influence of intermediate principal stress on the dynamic responses of rocks subjected to true triaxial stress state. Goodfellow [31] and Young [32] developed advanced systems primarily designed for applied research in geophysics and petroleum reservoir studies. Xu and Li [33,34] designed the first true triaxial apparatus in China, providing crucial experimental data for the development of the double-shear strength theory [35–37]. To comprehensively investigate the full stress-strain evolution and creep-induced rockburst characteristics of hard rock subjected to true triaxial stress, Feng [38] designed two overlapping Mogi-type high-pressure true triaxial apparatuses. Additionally, a true triaxial apparatus capable of time-dependent testing of hard rock under high-stress conditions was developed, featuring a main loading system with a two-rigid and one-flexible load approach [39]. Yin [40] developed a multifunctional true triaxial fluid-solid coupling experimental system to investigate the mechanical properties, seepage characteristics, and dynamic disaster features of reservoir rocks under true triaxial stress conditions. He [41,42] developed a large-scale true triaxial simulation test machine specifically for studying the rockburst process in hard rock working faces after roadway excavation. During the simulation of excavation-induced rockburst, the dynamic unloading cylinder quickly retracts, causing the swing arm to instantly drop. This exposes one or more free faces of the rock sample. This type of combined hydraulic true triaxial testing machine is also utilized in the TRW-3000 true triaxial hydraulic servo-controlled rock testing system at Central South University in China [43]. The large-scale true triaxial apparatus developed by Su [44,45] incorporates dynamic heterogeneous disturbance modes and rapid unloading capabilities. It is designed to study the degradation of mechanical properties in surrounding rock, potentially leading to engineering disasters such as rockbursts. This apparatus focuses on research related to excavation unloading simulation under asymmetric multi-axial stress states at the tunnel face. Additionally, scholars such as Wawersik and Frash [46–50] have developed true triaxial apparatus with unique functional characteristics.

(2) Dynamic impact true triaxial loading system

The Split Hopkinson Pressure Bar (SHPB) is a key technique and apparatus in rock dynamics experiments, capable of achieving strain rates up to 10^3^/s [51–54]. Building upon the true triaxial testing platform, a combined static and dynamic loading apparatus [55,56] was designed. This apparatus employs a 10 mm diameter disturbance bar with a localized disturbance area of 78 mm^2^, enabling it to simulate the scenario where an underground stope experiences overall static loading punctuated by localized dynamic disturbances. Xia [57] and Zhou [58] successively introduced axial static load and, subsequently, confining static load into the SHPB. The implementation involved placing the traditional SHPB in a system that provides axial reaction force support and using a Hugoniot pressure chamber to apply the confining static load. Experiments revealed that pre-applied static loads, both axial and confining, have a significant impact on rock strength characteristics, fragmentation, and energy behavior. Cadoni and Albertini [59] designed an SHPB apparatus capable of simulating biaxial or true triaxial stress states. The specimen size is a 60 mm cube, extendable to 100 mm. Both the incident and transmitted bars are 2 m in length, and the rectangular wave pulse force can reach up to 3000 kN. By recording dynamic strains with strain gauges, SHPB tests under bending and shear conditions can be conducted. Monash University in Australia developed a triaxial Hopkinson bar system [60] that enables impact testing of cubic specimens under three-dimensional static stress conditions. To investigate the mechanical behavior of engineering-disturbed rock masses in depth, the concept of engineering-disturbed rock dynamics was first proposed [61]. To deeply examine the mechanical behavior of engineering-disturbed rock masses, Xie [62] introduced the groundbreaking concept of engineering-disturbed rock mass dynamics and developed an advanced true triaxial electromagnetic Hopkinson system. This system marks a significant advancement in rock dynamic-static combined loading experiments, elevating the field to a new standard of precision and comprehension.

As previously highlighted, most existing true triaxial apparatus rely on hydraulic quasi-static loading methods, whereas dynamic rock mechanics experiments predominantly focus on the effects of high strain rate loading. Nevertheless, in deep rock mass engineering, a substantial portion of dynamic disturbance is characterized by low-frequency periodic dynamic loads. For instance, stress waves induced by distant blasting operations and seismic waves triggered by the instability of large rock masses near excavated cavities represent the primary sources of these loads. These low-frequency periodic disturbance loads are distinguished by their long propagation distances and high energy intensities. The mechanisms through which they induce deformation and failure in high-stress surrounding rock remain insufficiently understood. Therefore, investigating the mechanical behavior of rocks under the combined influence of high-stress and low-frequency dynamic effects, which can result in deformation and failure of surrounding rock masses is of critical importance.

To address the challenge of external dynamic disturbances affecting deep rocks under high-stress conditions, a novel true triaxial testing device, termed as the high-stress, low-frequency dynamic true triaxial apparatus (HS-LFD-TTA), has been developed. This apparatus independently and synchronously controls both dynamic disturbances and confining pressure. The paper primarily focuses on the structural components and key functionalities of the HS-LFD-TTA, while validating its stability and reliability through true triaxial static and disturbance tests conducted on granite.

## 2. Design and key parameters of the novel true triaxial apparatus

**Fig 1:**
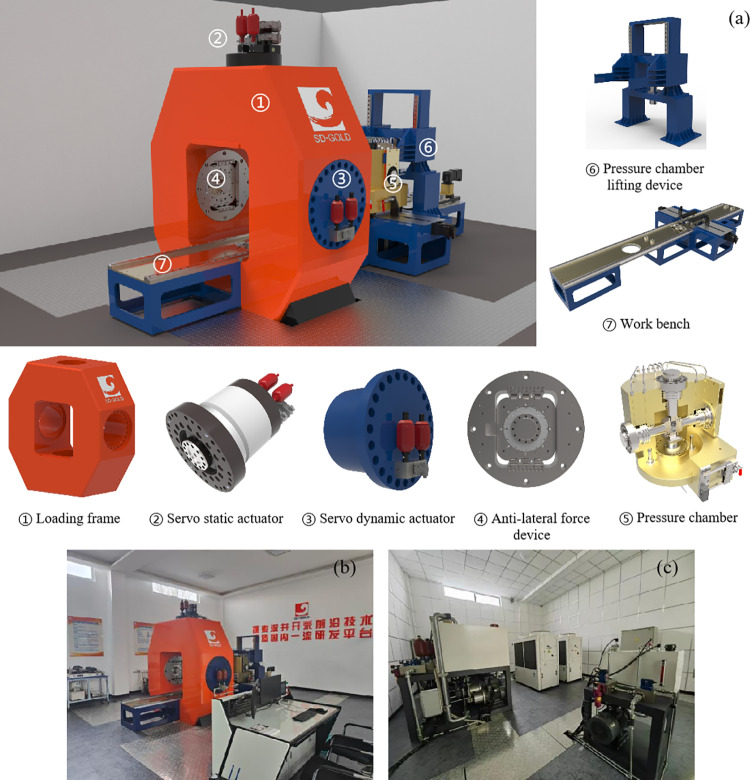
The high-stress, low-frequency dynamic true triaxial apparatus, (a) apparatus layout diagram, (b) host system and control system, and (c) hydraulic system and cooling system.

The HS-LFD-TTA is based on Mogi’s design concept, with optimizations to its mixed loading mode. It primarily consists of the following components: the host system, control system, hydraulic system, cooling system, and expandable modules, such as an acoustic emission acquisition system and a seepage loading system, as illustrated in Fig 1. Below are the main technical parameters of the system.

The HS-LFD-TTA is designed to simulate the in-situ stress environment at a burial depth of 2500 meters, enabling full stress-strain tests under high-pressure true triaxial static and dynamic loading conditions. Its primary objective is to identify the critical instability mechanisms under true triaxial conditions. The main technical parameters of the apparatus are as follows.

(1) Rock specimen size: 50 × 50 × 100 mm^3^
(2) Maximum load in the direction of the first principal stress: 2000 kN
(3) Maximum load in the direction of the second principal stress: 4000 kN
(4) Maximum stress in the third principal stress direction: 120 MPa
(5) Maximum response frequency of a low-friction actuator with normal stiffness: 20 Hz
(6) Deviation between the specimen’s geometric center and the load center: 0.05 mm
(7) Overall frame stiffness: 18 GN/m
(8) Hydraulic servo response time: 0.2 ms
(9) Sensor accuracy: 0.2% of full scale

## 3. Core technologies of the novel true triaxial apparatus

### 3.1 High-rigidity biaxial loading frame

**Fig 2:**
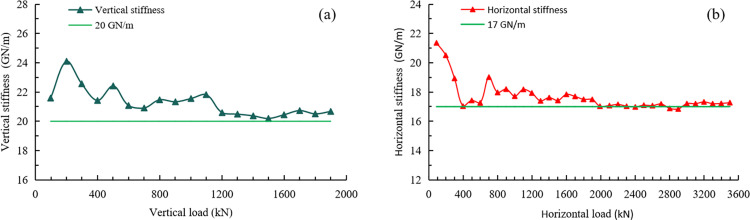
Stiffness test results of the frame, (a) vertical stiffness, and (b) horizontal stiffness.

The loading frame is the most essential hardware component of the HS-LFD-TTA. The stiffness of the frame plays a critical role in ensuring the stability of the experimental process and the accuracy of the post-peak curve. To meet the stringent requirements of true triaxial testing for deep rock, a high-stiffness loading frame structure with an orthogonal stress arrangement in both the horizontal and vertical planes has been proposed. The frame is integrally forged from 42CrMo high-strength alloy steel. Moreover, the actuators are capable of independent and unrestricted movement in both the horizontal and vertical directions of the loading frame. Tests have shown that the stiffness of the frame is at least 20 GN/m in the horizontal direction and at least 17 GN/m in the vertical direction, with minimal deformation. This fully meets the experimental requirements for equipment stiffness, as shown in Fig 2.

### 3.2 High-precision servo-controlled loading system

The HS-LFD-TTA is a representative example of a true triaxial testing machine that adopts a “two rigid and one flexible” design. The loading systems within this machine, oriented along the directions of the maximum, intermediate, and minimum principal stresses, operate independently without interference. Furthermore, each of these three directions is capable of closed-loop loading and unloading.

(1) Rigid loading system

**Fig 3:**
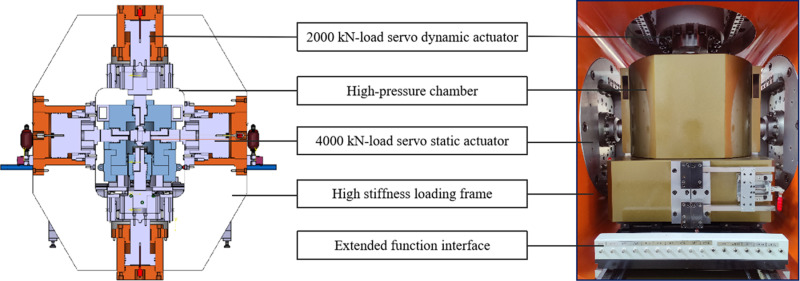
Schematic diagram of the rigid loading system.

**Fig 4:**
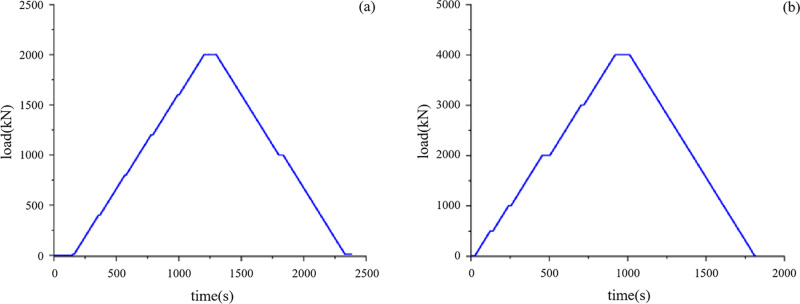
Maximum load test curve of the actuator, (a) dynamic actuator, and (b) static actuator.

**Fig 5:**
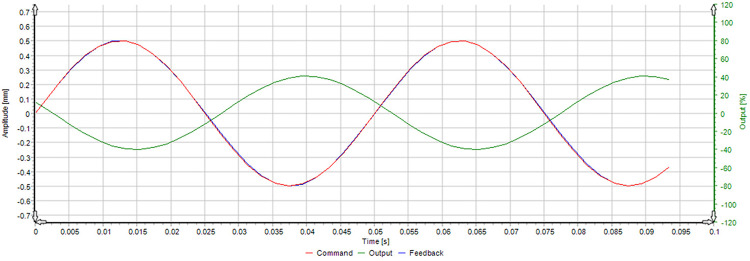
Testing of dynamic actuator disturbance amplitude and frequency.

Rigid loading of the maximum and intermediate principal stresses is achieved through the actuators, as shown in Fig 3. Specifically, the vertical direction is defined as the direction of maximum principal stress and is loaded by two dynamic servo actuators with high-frequency response, positioned above and below. Each actuator has a maximum load capacity of 2000 kN, as illustrated in Fig 4(a). Furthermore, this vertical direction is capable of generating low-frequency surface disturbances, with a maximum frequency of 20 Hz and an amplitude of 0.5 mm, as shown in Fig 5. In contrast, the horizontal direction is defined as the direction of intermediate principal stress and is loaded by two static servo actuators, positioned on the left and right, each with a maximum loading capacity of 4000 kN, as illustrated in Fig 4(b).

Controlled by the Doli controller, four high-pressure precision servo-loading pumps provide power to the actuators. The system is also equipped with Moog high-frequency response servo valves and accumulators, ensuring precise stress control during various stress path experiments and dynamic loading procedures.

(2) Flexible loading system

**Fig 6:**
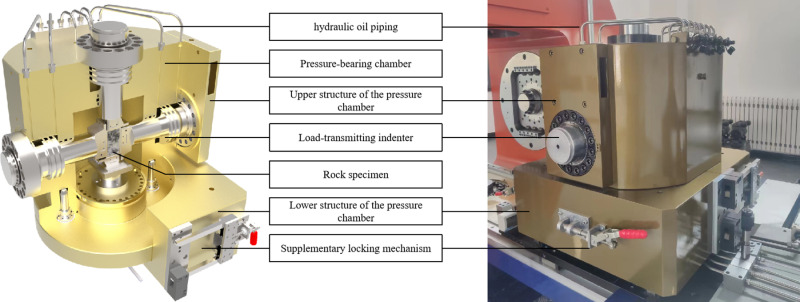
Structural diagram and photograph of the pressure chamber.

Flexible loading of the minor principal stress is achieved through hydraulic oil within the pressure chamber. The structure of the pressure chamber is shown in Fig 6. Four force-transmitting pistons are positioned within the vertical plane of the chamber to ensure compatibility with the biaxial test frame and to meet the conditions required for true triaxial testing. Featuring a self-balancing structure, the four sets of pistons ensure precise and stable movement of the pressing head during the loading of maximum and intermediate principal stresses, remaining unaffected by the confining pressure. Designed for a rated working pressure of 80 MPa and a maximum limit pressure of 120 MPa, as shown in Fig 6, the pressure chamber is equipped with a pressure sensor to enable real-time pressure monitoring, ensuring operational safety and reliability. The four sets of pistons, featuring a self-balancing structure, ensure precise and stable movement of the pressing head during the loading of the maximum and intermediate principal stresses, remaining unaffected by the confining pressure. Designed for a rated working pressure of 80 MPa and a maximum pressure limit of 120 MPa, the pressure chamber is equipped with a pressure sensor for real-time pressure monitoring, ensuring operational safety and reliability.

### 3.3 Precision measurement technology for triaxial deformation of rock

During true triaxial testing, it is essential to independently measure rock deformation along three orthogonal axes. To overcome the limitations of strain gauge-based measurement techniques and mitigate the influence of temperature fluctuations on measurement accuracy, a deformation measurement device based on the principle of Linear Variable Differential Transformers (LVDT) has been developed.

**Fig 7:**
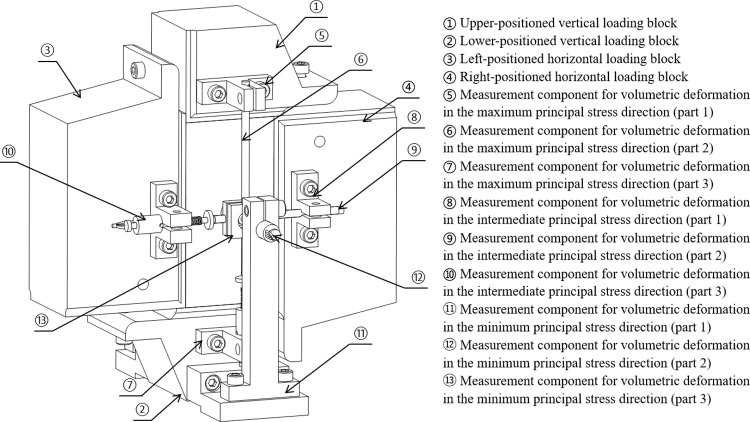
Three-directional deformation measurement device.

The deformation measurement devices consists of three components: a disc spring seat, connecting rods, and the LVDT sensor body. To measure deformation in the maximum and intermediate principal stress directions, the devices are installed on the upper and lower vertical loading blocks, as well as on the left and right horizontal loading blocks, respectively. For measuring deformation in the minimum principal stress direction, the devices are positioned on the front and rear of the lower vertical loading block. The connecting rods in all three directions are arranged perpendicularly in a “cross” configuration, ensuring they do not make contact with each other, thereby forming three independent measurement systems, as shown in Fig 7.

Since the deformation caused by hard rock failure typically does not exceed 1 millimeter, it is essential to choose a Linear Variable Differential Transformer (LVDT) with a narrow measuring range, high accuracy, and the ability to withstand high pressure. After conducting a comparative analysis, the LVDT-CD-375 series from Macro Sensors USA, which offers a measuring range of 1.2 millimeters and an accuracy of up to 0.05%, was ultimately selected. To prevent magnetic interference, the core of the LVDT sensor must be mounted on a precision-machined aluminum guide rod, ensuring the contact end surface remains perfectly smooth. Additionally, spring support ensures stable contact between the pressure block and the surface during sliding, thereby safeguarding the precision of the measurements.

### 3.4 Techniques for suppressing eccentric loading effects

**Fig 8:**
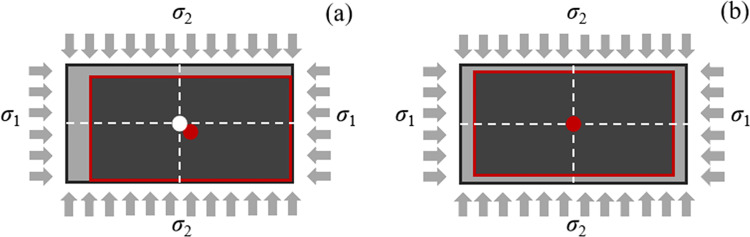
A rock specimen without (a) and with (b) the eccentric loading suppression technique. The white and red dots represent the initial and final positions of the specimen’s center, respectively.

In a conventional triaxial compression test, the specimen is fixed at one end, while the other end is compressed by an actuator. During the compression process, the specimen’s center often shifts, as shown in Fig 8(a). This undesirable phenomena, resulting from asymmetrical surface deformation, introduces additional shear stress and reduces the accuracy of the test. Ideally, the center should remain fixed as shown in Fig 8(b).

There are two approaches to addressing the issue of eccentric loading: one involves using a movable loading frame to keep the specimen centered throughout the test, while the other employs a dual-actuator control system to simultaneously apply symmetric loads, thereby minimizing the eccentric loading effect.

This paper adopts the second method, employing a Doli controller to regulate a set of actuators in each stress loading direction. The key to this approach lies in ensuring that the actuators operate synchronously under precise control, generating identical displacements and effectively eliminating the effects of eccentric loading.

The procedure is outlined as follows: (1) When initiating the loading of the principal stress, the computer sends a command to the Doli controller, which converts it into control signals for the loading pump rate according to the specified loading parameters. (2) Upon receiving the synchronous control signal from the Doli controller, the high-pressure servo loading pump begins to supply equal oil pressure to the piston for principal stress loading. The two oil cylinders in the same principal stress-loading direction are identical in specifications and, driven by the high-pressure servo loading pump, deliver equal displacements, ensuring a synchronous loading process.

**Fig 9:**
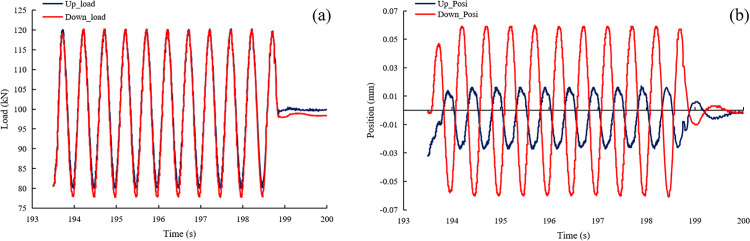
Testing curve for synchronous loading of dynamic actuators, (a) loading consistency test, and (b) displacement synchronization test.

**Fig 10:**
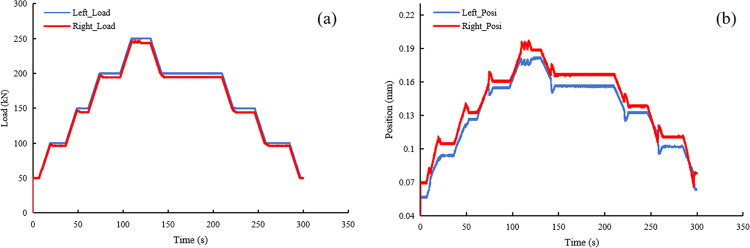
Testing curve for synchronous loading of static actuators, (a) loading consistency test, and (b) displacement synchronization test.

To verify the reliability of the loading system, LVDT sensors were employed to assess the loading consistency and displacement synchronicity of both the dynamic actuator (in the direction of maximum principal stress) and the static actuator (in the direction of intermediate principal stress). Fig 9 presents the results of a synchronization test for the dynamic actuators at a frequency of 2 Hz and an amplitude of 20 kN. The data show that the load value of the upward actuator stabilizes at 20 kN, while the downward actuator stabilizes at 21 kN, indicating good load consistency. Regarding displacement synchronization, the maximum displacement difference between the two actuators at corresponding time points is 0.05 mm. Fig. 10 presents the experimental results of a synchronization test for static actuators. In terms of load consistency, the loading curves of the two actuators nearly overlap, indicating excellent synchronization. Regarding displacement synchronization, the maximum displacement difference is 0.03 mm.

Based on the above analysis, it can be concluded that synchronization under static loading conditions is superior to that under dynamic loading. However, whether under dynamic loading (with a maximum center deviation of 0.05 mm) or static loading (with a deviation of approximately 0.03 mm), both values are small compared to the millimeter-scale deformation of the rock specimen. This indicates that the synchronous loading technique is highly effective in mitigating eccentric loading issues.

## 4. Verification test and result analysis

To assess the performance of the novel true triaxial apparatus, rock mechanics tests were conducted on granite under static and low-frequency disturbances. The rock samples used in these tests were collected from the -1005 m level of Sanshandao Gold Mine, located in Shandong Province, China. The samples have size of 50 × 50 × 100 mm^3^, and their processing accuracy conforms to the standards set by the International Society for Rock Mechanics and Rock Engineering (ISRM).

### 4.1 True triaxial static test

#### 4.1.1 Loading path.

**Fig 11:**
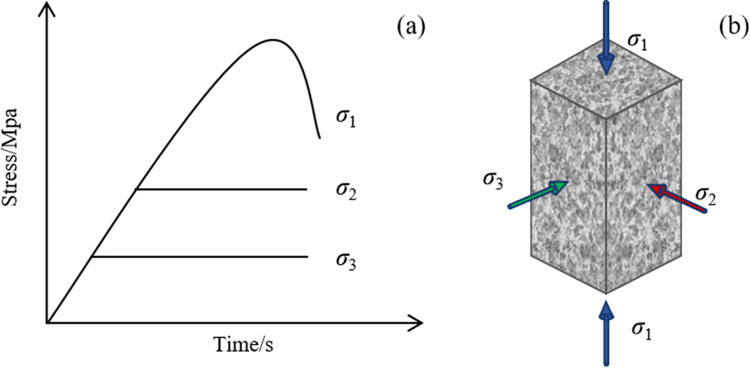
The loading path in a true triaxial static test, (a) loading stress path, and (b) loading status diagram.

The loading path for a static true triaxial test is shown in Fig 11. (1) Minimum principal stress loading stage: Hydrostatic pressure is applied uniformly (*σ*_1_ = *σ*_2_ = *σ*_3_) until the preset value (*σ*_3_) is reached. (2) Intermediate principal stress loading stage: The stress in the *σ*_3_ direction is maintained constant, while the stresses in the *σ*_2_ and *σ*_1_ directions are simultaneously increased to the preset value (*σ*_2_). (3) Maximum principal stress loading stage: With the stresses in the *σ*_2_ and *σ*_3_ directions maintained constant, continuous loading is applied to *σ*_1_ until specimen failure occurs. Initially, stress control is employed for loading; however, as the peak strength of the specimen is approached, the loading method transitions to deformation feedback control along the direction of the minimum principal stress to capture the full stress-strain curve of the rock.

#### 4.1.2 Results and analysis.

The peak failure strengths of granite were determined through a series of tests conducted under conditions of constant minimum principal stress while varying the intermediate principal stress, as summarized in Table 1. The minimum principal stress values were maintained at 0 MPa, 20 MPa, and 30 MPa, whereas the intermediate principal stress was systematically varied at 0 MPa, 50 MPa, 60 MPa, and 70 MPa.

**Table 1:** The peak failure strength of granite under different principal stress.

Number	*σ*_3_ (MPa)	*σ*_2_ (MPa)	*σ*_1,peak_ (MPa)	Average *σ*_1,peak_ (MPa)
1-1	0	0	157.5	162.8
1-2	0	0	168.1	
2-1	20	50	266.2	275.9
2-2	20	50	285.6	
3-1	30	50	326.4	318.75
3-2	30	50	311.1	
4-1	30	60	482.7	474.35
4-2	30	60	466.0	
5-1	30	70	382.4	394.8
5-2	30	70	407.2

(1) Analysis of stress-strain curves

**Fig 12:**
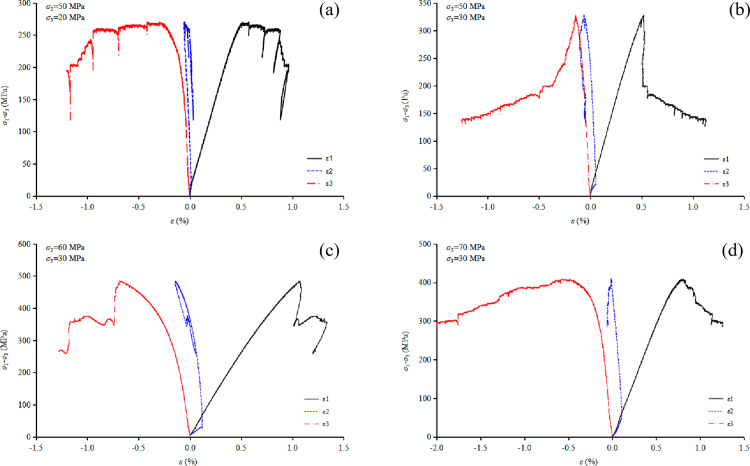
Stress-strain relationship curve for granite under true triaxial loading, (a) *σ*_2_ = 50 MPa, *σ*_3_ = 20 MPa, (b) *σ*_2_ = 50 MPa, *σ*_3_ = 30 MPa, (c) *σ*_2_ = 60 MPa, *σ*_3_ = 30 MPa, and (d) *σ*_2_ = 70 MPa, *σ*_3_ = 30 MPa.

From Fig 12(b) to Fig 12(d), it can be observed that the stress-strain behavior of granite under true triaxial compression varies with different values of *σ*_2_. When *σ*_3_ is maintained constant at 30 MPa, and *σ*_2_ is set to 50 MPa, 60 MPa and 70 MPa, the corresponding peak strengths (*σ*_1_) of granite are 326.4 MPa, 482.7 MPa, and 407.2 MPa, respectively. Similarly, the peak strains (*ε*_1_) are 0.63%, 0.91%, and 0.84%, respectively. These results demonstrate that both the failure strength and peak strain of granite under true triaxial loading initially increase and then decrease as *σ*_2_ increases, aligning with findings from previous research studies. Furthermore, as *σ*_2_ increases, the divergence between the *σ*_1_-*ε*_2_ curve and the *σ*_1_-*ε*_3_ curve widens significantly, highlighting the critical influence of *σ*_2_ on the differential deformation of the rock. Specifically, the greater the difference between *σ*_2_ and *σ*_3_, the larger the disparity in deformation between the *ε*_2_ and *ε*_3_ directions under true triaxial stress conditions.

A similar conclusion can be drawn from Fig 12(a) to Fig 12(b), namely, that both the failure strength and peak strain of the rock increase with increasing *σ*_3_ under true triaxial stress conditions. From the shape of the stress-strain curves, it can be observed that as *σ*_3_ increases, the rock exhibits a tendency towards Type I failure. This suggests that an increase in *σ*_3_ reduces the brittle failure characteristics of the rock. Furthermore, when *σ*_3_ increases from 20Mpa to 30 MPa, the failure strength increases by 40.8 MPa, whereas when *σ*_2_ increases from 50 Mpa to 60 MPa, the failure strength increases by 156.3 MPa. This indicates that the influence of *σ*_3_ on the strength of granite is somewhat less pronounced than that of *σ*_2_.

(2) Analysis of failure modes

**Fig 13:**
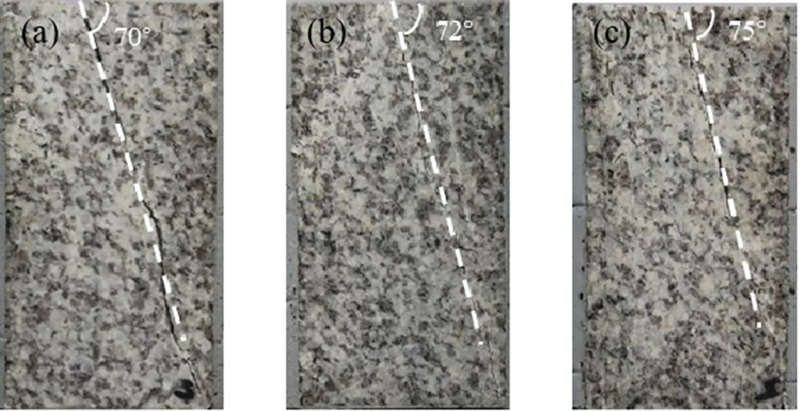
Failure mode and fracture angle of granite under true triaxial compression, (a) *σ*_2_ = 50 MPa, (b) *σ*_2_ = 60 MPa, and (c) *σ*_2_ = 70 MPa.

As shown in Fig 13, most of the samples exhibit a single macro-scale main crack, with the failure mode of granite primarily characterized by shear failure. A relatively larger number of macro-cracks are observed on the *σ*_1_-*σ*_3_ plane.

Many previous studies have investigated the variation in the rock fracture angle with change in *σ*_2_. For example, Haimson et al. proposed that the fracture angle of granite increases monotonically with an increase in *σ*_2_ with *σ*_3_ held constant. In contrast, Feng et al. suggested that under true triaxial stress conditions, the fracture angle of rock initially increases and then decreases as *σ*_2_ increases. Within the stress levels discussed in this paper, when *σ*_3_ is 30 MPa, the fracture angle of granite increases significantly with the increase in *σ*_2_ exhibiting a clear trend.

### 4.2 True triaxial low-frequency disturbance test

#### 4.2.1 Loading path.

**Fig 14:**
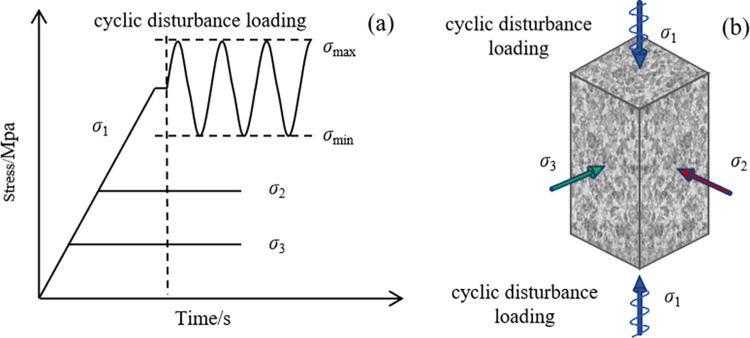
The stress path in true triaxial disturbance test, (a) loading stress path, and (b) loading status diagram.

The loading path comprises two stages: the static loading phase and the dynamic disturbance loading phase, as shown in Fig 14. The static loading phase mirrors the procedures used in the true triaxial static test. During the dynamic disturbance loading phase, the specimen is subjected to a predetermined target stress level, as specified in the test protocol. Once the desired static stress state is established, sinusoidal dynamic disturbance loads are applied to the specimen.

The disturbance loading is applied using displacement control, with the lower stress set to the static stress in the *σ*_1_ direction, while the upper stress is maintained below the average peak strength obtained from true triaxial static tests. If the specimen does not fail after cyclic disturbance loading, deformation feedback control is implemented along the minimum principal stress direction to continue loading until specimen failure occurs.

#### 4.2.2 Results and analysis.

**Fig 15:**
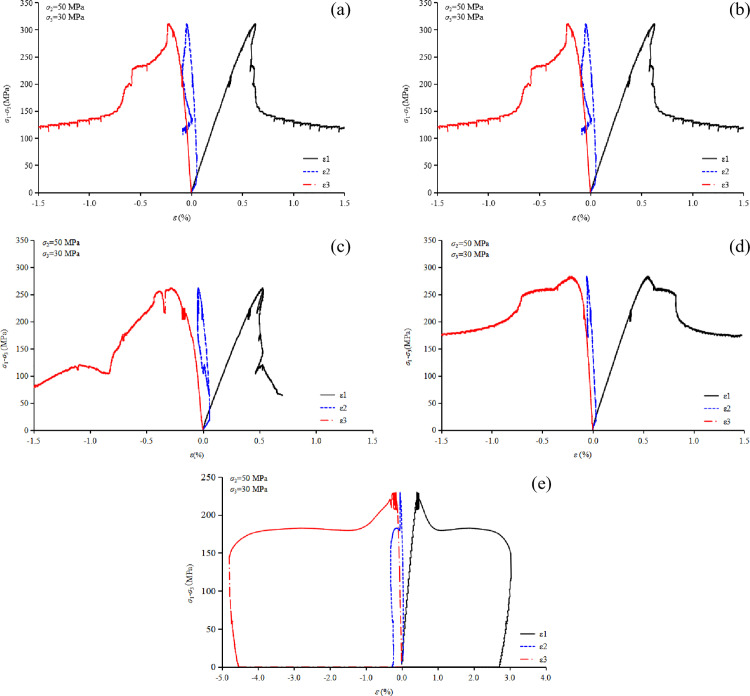
Stress-strain relationship curve for low-frequency disturbance tests, (a) 4 Hz 30 kN, (b) 6 Hz 30 kN, (c) 8 Hz 30 kN, and (d) 10 Hz 30 kN.

The minimum and intermediate principal stresses were set to 30 MPa and 50 MPa, respectively. Disturbance loading with an amplitude of 12 MPa was applied at frequencies of 4 Hz, 6 Hz, 8 Hz, 10 Hz and 12 Hz when the axial stress reached 80% of the average static peak strength, with each frequency corresponding to a total of 2000 disturbance cycles. If the rock specimen did not fail under these dynamic loading conditions, axial static loading was resumed until a full stress-strain curve was obtained. The stress-strain curves resulting from the low-frequency disturbance tests are illustrated in Fig 15.

(1) Analysis of stress-strain curves

Fig 15 illustrates the stress-strain curves obtained from the low-frequency disturbance tests. The loading process consists of two stages: an initial static loading phase to reach the target stress level, followed by the application of low-frequency cyclic dynamic disturbances with an amplitude of 12 MPa. During the cyclic disturbance phase, the maximum principal strain accumulates progressively as the number of cycles increases. Failure occurs only when the maximum principal strain reaches the specimen’s ultimate failure threshold. When *σ*₂, *σ*₃, and the vibration amplitude held constant, an increase in disturbance frequency results in a reduction in the specimen’s peak strength at failure. Specifically, at disturbance frequencies of 4 Hz, 6 Hz, 8 Hz, and 10 Hz, the specimen remains intact under the applied cyclic load, while its peak failure strength decreases as the frequency increases. In contrast, at a frequency of 12 Hz, the rock fails during the disturbance phase, indicating that this frequency exceeds the specimen’s stability threshold.

In the analysis of peak strength, with the minimum and intermediate principal stresses set at 30 MPa and 50 MPa, respectively, the average peak strength of the rock in the static test was measured as 318.75 MPa. Under the same stress conditions, with a disturbance amplitude fixed at 30 kN and disturbance frequencies of 4 Hz, 6 Hz, 8 Hz, 10 Hz, and 12 Hz, the corresponding peak strengths of the rock were recorded as 314.1 MPa, 299.8 MPa, 282.7 MPa, 261.8 MPa, and 229.4 MPa, respectively. These results indicate that the reduction in peak strength becomes increasingly significant as the disturbance frequency rises, with decreases of 1.46%, 5.95%, 11.31%, 17.87%, and 28.02%, respectively.

For low-frequency disturbances (4 Hz and 6 Hz), the reduction in peak strength is relatively minor, indicating that such disturbances have a limited impact on the structural integrity of the rock. However, at frequencies of 8 Hz and higher, the rate of strength reduction accelerates substantially. At 12 Hz, the specimen fails during the disturbance process, emphasizing the significant weakening effect of high-frequency disturbances on the rock’s structural stability. From the perspective of curve morphology, as the disturbance frequency increases, the maximum principal strain at failure decreases. Consequently, the full stress-strain curve transitions gradually from Type II to Type I, illustrating the evolution of the rock’s failure behavior under dynamic loading conditions.

These phenomena are likely closely associated with the rapid expansion of microcracks induced by the disturbance and the mechanisms of internal energy accumulation and release within the rock. Specifically, the disturbance exacerbates stress concentration and accelerates crack propagation, resulting in a marked reduction in the rock’s peak strength.

(2) Analysis of failure modes

**Fig 16:**
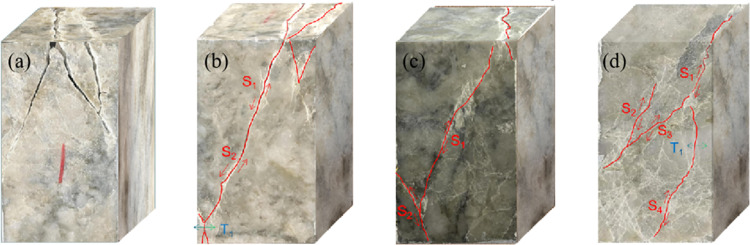
The failure modes of rock specimens, (a) 0 Hz 0 kN, (b) 4 Hz 30 kN, (c) 8 Hz 30 kN, and (d) 12 Hz 30 kN.

Fig 16 illustrates the failure modes of specimens under static loading and three distinct frequencies of dynamic disturbance. Under static loading conditions, the specimens primarily exhibit shear failure, characterized by cracks parallel to the *σ*₁ loading direction in the vicinity of the *σ*₃ regions, accompanied by splitting cracks also aligned with the loading direction. Prominent shear cracks are observed in the central region of the specimen, leading to localized fragmentation and significant expansion. These failure modes exhibit a multi-regional pattern, where the specimens retain partial load-bearing capacity even after failure.

However, under low-frequency disturbance loads, the multi-regional failure characteristics of the specimens become increasingly pronounced. Central cracks tend to fully penetrate the specimens, and the number of cracks induced by dynamic disturbances increases with rising disturbance frequency. Moreover, the specimens exhibit significant expansion and localized fragmentation, resulting in more extensive and severe failure. These observations underscore the critical role of dynamic disturbances in intensifying the failure of high-stress rocks, particularly under low strain-rate conditions.

## 5. conclusion

A novel true triaxial apparatus (HS-LFD-TTA) has been successfully developed at the Shandong Gold Deep Mining Laboratory in China to address the challenges of rock mechanics in deep underground environments, characterized by high-stress conditions and low-frequency disturbances. This paper provides a detailed and comprehensive overview of the apparatus’s functional capabilities, core technological advancements, and verification tests. The main conclusions are summarized as follows:

(1) A high-rigidity biaxial loading frame was designed, with horizontal and vertical stiffness values of 20 GN/m and 17 GN/m, ensuring that the system meets the stiffness requirements for obtaining full stress-strain curves and controlling post-peak behavior. A two-rigid and one-flexible loading method, combined with a servo control system, enabled precise stress control in all three loading directions.
(2) An eccentricity suppression technique was proposed, utilizing synchronous dual-actuator loading to maintain the deviation between the specimen’s geometric and load centers within 0.05 mm, effectively eliminating eccentric loading in true triaxial testing. A deformation measurement system based on the principle of linear variable differential transformers (LVDT) was designed, overcoming the limitations of traditional strain gauges and enabling independent, precise deformation measurement in all three directions.
(3) The HS-LFD-TTA features combined static and dynamic synchronous loading. Static loads are applied in the intermediate and minimum principal stress directions, while dynamic actuators impose uniform disturbance loads in the maximum principal stress direction, with a maximum frequency of 20 Hz and amplitude up to 0.5 mm. This enables precise simulation of low-frequency disturbance effects on deep rock masses.
(4) True triaxial static and disturbance tests on granite revealed that both the intermediate principal stress and disturbance frequency significantly influence rock strength and failure modes. The tests exhibited good repeatability and consistency, validating the apparatus’s accuracy and reliability. The successful development of the HS-LFD-TTA offers a novel approach for studying deep rock mechanics under high-stress, low-frequency disturbance conditions, providing an effective tool for scientific and engineering challenges.
